# Neural Signatures of the Configural Superiority Effect and Fundamental Emergent Features in Human Vision

**DOI:** 10.1038/s41598-018-32289-2

**Published:** 2018-09-17

**Authors:** Thiago Leiros Costa, Kimberley Orsten-Hooge, Gabriel Gaudêncio Rêgo, Johan Wagemans, James R Pomerantz, Paulo Sérgio Boggio

**Affiliations:** 1 Laboratory of Experimental Psychology – KU Leuven, Leuven, Belgium; 2 Social and Cognitive Neuroscience Laboratory, Mackenzie Presbyterian University, São Paulo, Brazil; 3 Department of Psychology, Rice University, Houston, USA; 4 Department of Psychology, UT Dallas, Dallas,TX, USA

## Abstract

The concepts of grouping, emergence, and superadditivity (when a whole is qualitatively different from the sum of its parts) are critical in Gestalt psychology and essential to properly understand the information processing mechanisms underlying visual perception. However, very little is known about the neural processes behind these phenomena (particularly in terms of their generality vs. specificity and their time-course). Here, we used the configural superiority effect as a way to define “emergence” and “emergent features” operationally, employing an approach that can isolate different emergent features and compare them on a common scale. By assessing well-established event related potentials in a HD-EEG system, we found that the critical processes behind configural superiority and superadditive Gestalt phenomena are present in the window between 100 and 200 ms after stimulus onset and that these effects seem to be driven by specific attentional selection mechanisms. Also, some emergent features seem to be differentially processed in different brain hemispheres. These results shed new light on the issues of the generality vs. specificity of the neural correlates of different Gestalt principles, the hemispheric asymmetries in the processing of hierarchical image structure and the role of the N1 ERP component in reflecting feature selective mechanisms.

## Introduction

Early Gestalt psychology has offered valuable insights into how perceptual experiences are intrinsically holistic and generally seamless, proposing a number of principles of perceptual organization, frequently coupled with compelling behavioral demonstrations. The idea that a perceptual “whole” is different from what could be predicted by summing its constituent parts is still central to current vision science. But operationalizing some of the traditional Gestalt principles, assessing their neural mechanisms and most critically, integrating this knowledge into an explanatory theoretical framework has proven to constitute an outstanding challenge^1,2^. We argue here that the classical Configural Superiority Effect (CSE, described in Pomerantz, Sager and Stoever^3^) can be used to make progress in this context, as it may serve as a tool for defining Gestalts operationally, allowing us to assess their neural correlates and their generality (a critical step forward towards a theory of perceptual organization). The current work will focus on the neural correlates of the CSE, while also assessing the generality vs. specificity of the neural correlates of some critical and fundamental Gestalt principles.

### Configural Superiority and Emergent Features in Vision

**Figure 1:**
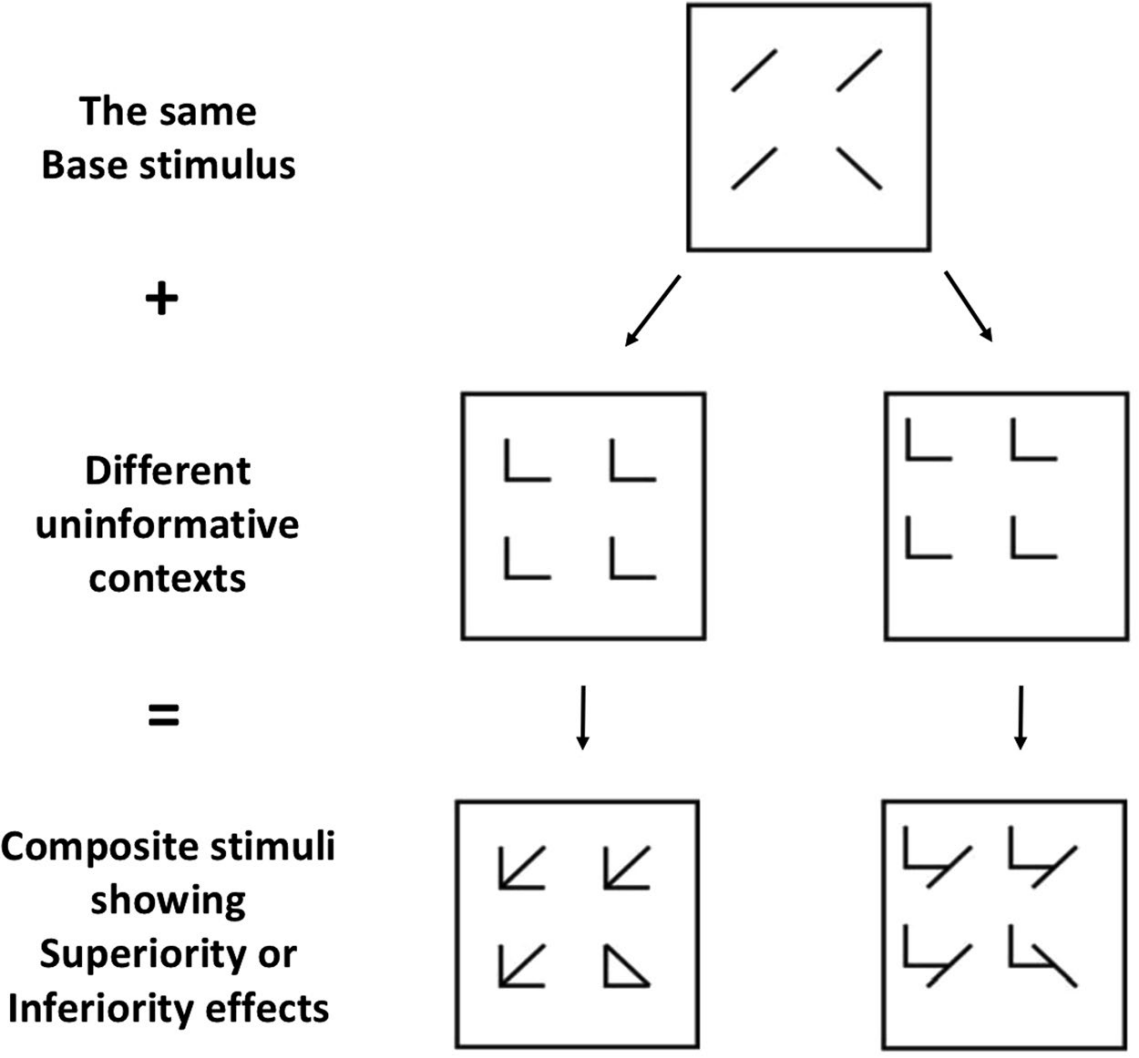
Examples of the classic displays used to introduce the Configural Superiority Effect (CSE). The same base display (top) coupled with redundant uninformative contexts (middle) will induce a CSE (bottom left) when this combination leads to emergent features (such as closure, number of terminators, and intersections). When no such emergent features are present (right), no CSE will be found; rather, Inferiority.

If discrimination of elements is aided by adding redundant uninformative context, we say that a Configural Superiority Effect has happened (see Fig. 1 for a classical example). The effect is robust and has been replicated in a number of circumstances, including other species (e.g., present in chimpanzees^4^ but absent in rats^5^). However, only few possible contexts will lead to a CSE. In fact, adding uninformative context should intuitively increase processing load along with increasing clutter and crowding, leading to slower and less accurate responses. But when different features in the stimulus and context interact in a way that will cause a whole to be perceived as something other than the sum of its parts, new features emerge, together with a qualitatively different percept. These features, which we call *emergent features* (EFs^3,6^), are then the fundamental driving force behind the CSE^7^.

The CSE is a remarkable demonstration of the holistic nature of visual perception and the classic Gestalt motto of the whole being different from the sum of the parts. In this context, Pomerantz and Portillo^8^ proposed that it may also help us to define Gestalts operationally. Early Gestalt psychologists have proposed an extensive number of principles based mostly on visual demonstrations and missing operational definitions, something that has proven critical in moving the field forward^1,2^. Operationalizing these Gestalt principles in terms of their specific effects in discrimination performance could provide us with a common scale of measurement to compare them. Maybe not all Gestalt phenomena may be assessed like this, but basic grouping principles and EFs might. The idea is that in order to qualify as an EF, a certain feature must induce a CSE (see Fig. 1). With this in mind, Pomerantz and Portillo^8^ stripped down the classical lines and arrows stimuli (known to generate a CSE^3^) to isolate the most fundamental EFs possible. Starting with four single dots in an odd quadrant visual search task with only local demands (as these single dots should not lead to EFs) and gradually superimposing more context dots, the authors were able to successfully isolate multiple EFs: orientation, proximity, linearity and surroundedness (i.e., found specific combinations of these dots that led to a CSE). We use this approach here in Experiment 2.

This proposal of operational definitions of Gestalts as EFs leads to results that may also be described as classic grouping principles (i.e., the basic principles by which different elements may or may not be bound together as relevant perceptual units). Therefore, one can use this approach to compare different grouping principles on a common scale with highly compatible stimulus sets. Pomerantz and Portillo^8^ did so and found some differences in reaction times between stimulus displays with different EFs. But one may ask then, what are the fundamental EFs used by the human visual system? Are these the same in terms of cortical visual processing? Can it be that some of these may not reflect specific principles but rather a more overarching general mechanism of visual holistic processing?

It has been proposed that perception is organized hierarchically with numerous functional specializations and some went as far as to suggest perception is “a bag of tricks”^9^. When it comes to grouping and EFs, they could either reflect “one overarching principle” (e.g., simplicity, goodness or “Prägnanz”^2^) or “multiple tricks”. There seems to be evidence to support the former, as there have been suggestions of neural synchrony as a mechanism for perceptual binding^10,11^ (although not without controversy^12^) or of a shared circuitry for proximity and similarity grouping^13^. On the other hand, there also seems to be evidence for the latter, e.g.: good continuation and grouping by proximity acting independently when aligning collinear Gabors in pathfinder displays^14,15^, and reports of differences in the time course of activations of proximity and similarity grouping^16–18^, or similarity and collinearity^19^. In this same line of thought, a recent review by Wagemans^20^ supports the idea that “not all Gestalts are equal” based both on theory and empirical findings from contemporary visual neuroscience studies.

But if that is true, then, how and when do they differ? Relying only on discrimination accuracies, reaction times and processing capacity^8,21^ has not allowed us to answer this question so far. Experimental phenomenology^22^ also does not seem to suffice. Using stimuli limited in the number of grouping principles they can compare is also a massive constraint. We argue here that one way to approach this question is to assess the neural correlates of EFs using the dot displays used by Pomerantz and Portillo^8^.

### Neural correlates of Configural Superiority and other Gestalt phenomena

Current visual science still struggles with assessing neural correlates of Gestalt processes. One of the reasons for that is the challenge of defining Gestalts operationally and finding stimulus sets with minimal confounders. As a result, such studies are still somewhat scarce. For instance, although CSEs are replicable and robust demonstrations of the holistic quality of visual perception, the neural correlates of this effect are still not so clear. Experiments showing CSEs have been around since the 1970’s, but the first study to assess its neural correlates was published only recently^23^. By using fMRI and multivoxel pattern analysis, these authors have found a decoding advantage for the “parts” stimuli (single line displays) in the primary visual cortex and for the “wholes” stimuli (configural displays inducing CSE) in the shape-selective lateral occipital cortex (LOC) in the ventral visual stream. These results suggested that holistic or Gestalt aspects of visual perception (or at least the CSE) may emerge only at intermediate/high stages in the visual processing hierarchy. This functional specialization for the CSE at the LOC was supported by a case study of the visual agnosia patient DF, who suffered bilateral LOC lesions^24^. The patient showed a behavioral reversal of the CSE (i.e., better discrimination of parts than wholes) and a reversal of the fMRI results seen in Kubilius *et al*.^23^.

A more recent fMRI study has suggested that the emergence of the CSE in the visual processing hierarchy is not an “all or nothing” phenomenon. By using displays with multiple degrees of configurality that ranged from not inducing to inducing CSEs and a more traditional univariate analysis of BOLD fMRI responses, Fox *et al*.^25^ found CSEs to emerge gradually in the visual processing hierarchy. More configural stimuli gradually led to stronger CSE and these changes in behavior were significantly correlated to changes in brain activation at multiple steps (as early as V1). But it is important to note that stronger BOLD responses in V1 does not imply better decoding of CSE-generating stimuli in V1^23–25^.

The works mentioned above^23–25^ suggest different views of the emergence of the CSE in the hierarchy of cortical visual processing, but both agree that there is a qualitatively different state of brain activation when dealing with EFs. Here, we argue that it is not possible to have a clear view of the dynamics of such phenomena using neuroimaging methods with a temporal resolution limited to the range of seconds. Claiming that the context of a stimulus may affect how it is processed as early as V1 finds support in a body of literature of other phenomena like figure-ground segregation^26^. These effects were observed in the first 100 ms after stimulus onset. One must expect numerous iterations between different hierarchical processing steps to happen in a time window that may not be assessed through fMRI and hence, faster methods are required (see van Leeuwen, 2015, for a review of traces of holistic processing at many stages in the visual hierarchy^27^). To the best of our knowledge, no EEG or MEG work has assessed the issue of configural superiority to this date. In fact, knowledge of the neural correlates of configural Gestalts is so limited that even the issue of potential hemispheric asymmetries in the visual cortex or the potential attentional selection mechanisms behind it is still not clear. The issue of different EFs having potentially different neural correlates has also hardly been approached with the necessary level of detail, as discussed below.

### Limitations of previous studies and current goal

Most of the studies on the neural correlates of perceptual organization and grouping so far have approached the problem mainly with three strategies: (i) assessing the primacy of holistic processing^28^ (frequently with compound hierarchical stimuli, as Asch-Navon displays), (ii) by contrasting grouped vs. ungrouped stimulus patterns (as with pathfinder displays^29^), or (iii) by parametrically manipulating one or two grouping principles and assessing how brain activity changes in relation to it (as with grouping by proximity^30^). All these approaches mentioned above are limited in their ability to compare grouping principles and some may also induce significant confounders. For instance, pathfinder displays^14^ are always limited by grouping by collinearity or good continuation and may also induce substantial visual search and contour integration demands as confounders (as stimuli are embedded in a field of noise^31^). Bistable dot lattices (or Gabor lattices^32^) are also limited in the different types of grouping principles they may present (generally only proximity and some variation of grouping by similarity) and their bistable nature may lead to numerous confounders in a neuroimaging setup. Other classical stimuli used in this literature (as hierarchical compound displays) also suffer from the limitations mentioned here.

We argue then, that the cleanest way to assess the generality vs. specificity of the neural correlates of basic EFs is with the current approach: (i) defining EFs operationally based on their effects on discrimination performance; (ii) using stimuli and tasks that allow them to be compared on a common scale, and (iii) in a high-density EEG setup, providing the required high temporal resolution. But in order to apply this approach appropriately, one must first better understand the neural correlates of the CSE in terms of how it evolves over time and what the attentional allocation mechanisms behind it are. For that, we propose an exploratory EEG investigation of the neural correlates of the CSE, followed by an assessment of these when specific EFs are isolated. We hope to unveil any similarities or differences between the potential neural correlates of these EFs. We expect that stimuli generating CSE will lead to clear differences in the amplitude of visual event related potentials (ERPs) compared with stimuli that do not generate CSEs; that different EFs may lead to different EEG signatures (although it is hard to speculate a priori what these would be); and that neural correlates of attentional deployment will act differently for stimuli that induce a CSE (reflected in amplitudes or latencies) than for stimuli that do not.

## Methods

**Figure 2:**
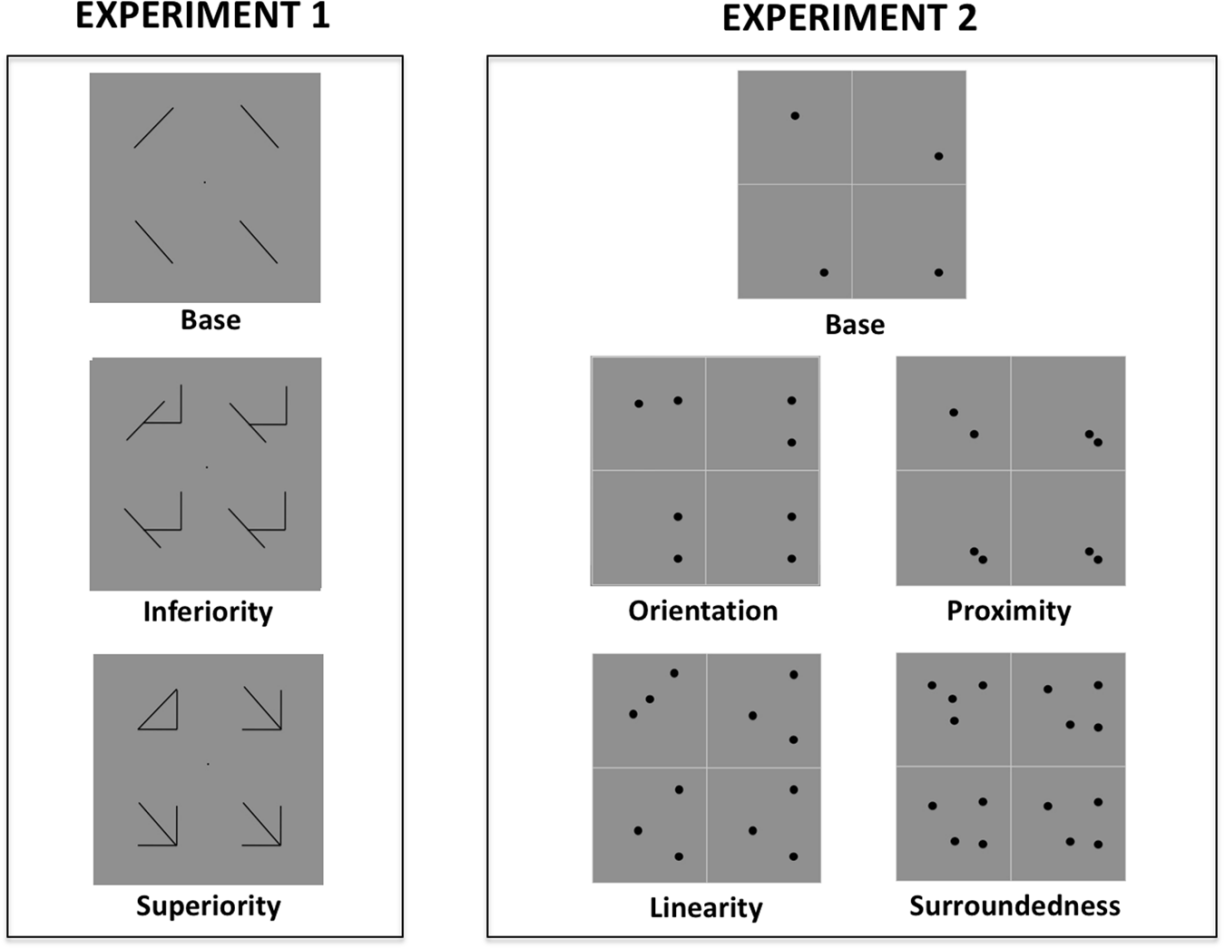
Stimuli used in Experiment 1 (left) and Experiment 2 (right). Both experiments included a base display upon which multiple context elements are added. In Experiment 1, one context leads to a CSE fx1while the other leads to a Configural Inferiority effect (CIE). In Experiment 2, the context elements added lead to different CSEs arising from specific EFs. We took the liberty to name the stimuli with the outcome of our measures (e.g. “Superiority” for CSE generating stimuli) given the existence of previously published and replicated results that showed these outcomes using very similar stimuli.

Two complementary experiments were performed. All procedures were the same in both, except for the stimuli used (see Fig. 2).

### Participants

In each experiment, we tested 16 healthy right handed subjects (23 ± 6 years, 8 male) who were naive to the stimuli and objectives of the study. All participants had normal or corrected to normal visual acuity and were screened for current medication use and any relevant neuropsychiatric alterations. All participants were adults and signed a written informed consent in accordance with local and international standards. The sample size and number of trials were decided based on what was proved to be effective in previous experiments from the literature and our own lab. All procedures were approved by CONEP, the Brazilian commission for ethics in scientific research (CAAE: 80576017.5.0000.0084).

### Stimuli

In both experiments, stimuli were black against a gray background with an average luminance of 80 cd/m^2^. Display sizes were always 7.5 × 7.5° dlegrees of visual angle. Each display had four stimuli - three identical distractors and one unique target. For all displays in Experiment 1, the only difference between the unique and the three identical quadrants was the orientation of one single line (45° in the unique and 135° in the identical ones). The same was true in Experiment 2, as the only difference between quadrants in every display was the location of one single dot. This allows the individual display types to be highly comparable in each experiment, as at the local level, the odd quadrant differs from the others in the very same local element.

In Experiment 1 we used the classic stimuli inducing CSE, called here “Superiority” (arrows and triangles known to induce strong CSE as they have multiple highly salient EFs), a similar control set called “Inferiority” (a control condition with the same lines and context as the stimuli inducing CSE, but missing the strong EFs and therefore not inducing CSE), and “Base” displays containing only single diagonal lines oriented 45° and 135°. These stimuli are basically the same used in Pomerantz *et al*. (1977)^3^ and almost the same as Kubilius *et al*.^23^ (except that the “Inferiority” condition was not tested there). In total, 12 unique displays were used (i.e., three types of displays x four possible locations of the odd element within the display).

In Experiment 2, we used dot displays based on what was used in Pomerantz and Portillo^8^. The quadrants of these displays were delimited by a low contrast, central crosshair. The displays are constructed from the ground up. The most basic one (“Base”) consists of single dots that differed only in their location within the quadrants. That constitutes the foundation over which all other displays were built. By adding one uninformative and redundant context dot to each quadrant, one can induce the EFs of “Proximity” and “Orientation” in isolation from each other, depending on the specific positions of the context dots. In a similar fashion, by adding two uninformative and redundant context dots to each quadrant, one can induce the EF of “Linearity”. Lastly, by adding more dots one can induce the EF of “Surroundedness” in which one dot falls inside the convex hull defined by the other three dots. In total, 20 unique displays were used (i.e., 5 types of displays x 4 possible locations of the odd element within the display).

### Procedure

In both experiments, subjects were tested in an odd-quadrant visual search task. Participants were informed that four elements would be presented in each display, three of these being identical and the fourth being different. Their task is to locate the quadrant that is different by pressing a remote control. At the start of each experimental session, the subjects would perform a version of the same task with mock stimuli (three black circles and one white circle) and accuracy feedback for 20 trials to get acquainted with the procedure. We used a design where only one single type of display was presented throughout a block. The order of the blocks in an experimental session was counterbalanced across participants. One hundred trials were presented for each block. For both experiments, stimuli were presented for 200 ms and preceded by a fixation dot presented for 500 ms. Participants had up to 1.7 seconds to make their responses.

### EEG recording and Analysis

Scalp EEG was continuously recorded from 128 active electrodes (with a Geodesic Sensor Net, EGI, Eugene-OR), digitized at a sampling rate of 1024 Hz, referenced to the Cz electrode and filtered online from 0.1 to 100 Hz. Recording and preprocessing of the data was done with Netstation 4.6 (EGI, Eugene-OR). Vertical and horizontal EOG were also recorded. Impedances were kept below 50 kΩ at all electrodes. Data was re-referenced offline with the average of all electrodes, downsampled to 250 Hz and a low-pass filter of 30 Hz was applied for the ERP analyzes. Analyzes focused on the first 500 ms after stimulus onset. EEG epochs were synchronized with the onset of the stimulus and cut with a baseline of 200 ms. Segments were baseline-corrected using this 200 ms prestimulus interval. Automatic artifact rejection was used, removing epochs for which peak-to-peak amplitudes exceeded 50 μV. Epochs with eye movements were also excluded. Participants had at least 70% good epochs per condition.

**Figure 3:**
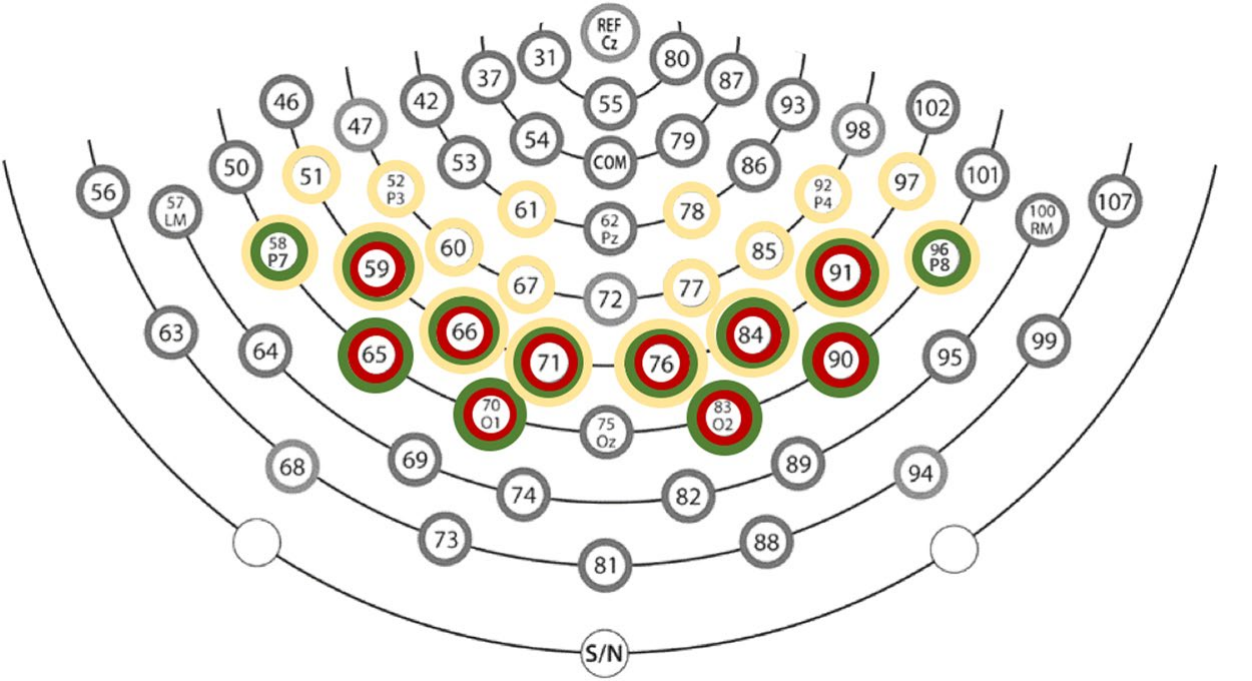
Schematic representations of the electrode clusters used in the analyses of the P1 (circled in red), N1 (in green) and the late positive component (in yellow), using the system of the Geodesic Sensor Net from EGI (Oregon - USA). Electrodes that coincide with the coordinates of the International 10–20 system are specified here for reference. For clarity, we plot only the back of the head here (i.e. from the vertex to the most posterior electrodes). The electrodes used for each component are marked in red and listed here. P1 Left: 59, 65, 66, 70(O1), 71. P1 Right: 76, 83(O2), 84, 90, 91. N1 Left: 58(P7), 59, 65, 66, 70(O1), 71. N1 Right: 76, 83(O2), 84, 90, 91, 96(P8). Late Positivity Left: 51, 52(P3), 58(P7), 59, 60, 61, 66, 67, 71. Late Positivity Right: 76, 77, 78, 84, 85, 91, 92(P4), 96(P8), 97.

In order to select the regions of interest for our analyzes of ERPs (both in space and time) we adopted a data-driven approach that was recently shown to not inflate Type I error rate^33^. The “Aggregate Grand Average From Trials” (AGAT) suggests that before selecting regions of interest, one must aggregate all trials from all participants and all conditions in a single data set and average this together for further assessment (e.g., in a single voltage map). Then, based on this average, one may select the electrode groups that show maximum voltages and the time windows where these effects happen. We created a data set with the AGAT and plotted multiple topographical voltage maps (at latencies 10 ms apart from stimulus onset) by interpolating voltages from different electrodes. There, we identified a bilateral cluster of activation around 100 ms at occipital sites, followed by a broader bilateral cluster in the negative polarity at lateral occipital sites at ~170 ms, followed by a positive occipito-parietal cluster peaking between 300 and 400 ms after stimulus onset. Then, we identified the peak voltage in each of these points and considered as a cluster the group of electrodes that deviated by up to 0.5 μV from that peak (this cluster selection procedure was derived from works such as Nikolaev *et al*.^30^, and others that have used high density EEGs). The specific electrodes used at each of these time windows are shown in Fig. 3. Lastly, single trials were averaged at the level of subject and condition, leading to typical ERPs. This left us with typical P100 and N170 occipital components, followed by a widespread occipito-parietal positive component peaking between 300 and 400 ms after stimulus onset^34^.

One exception to this approach was the N2pc component. It is a well-established neural correlate of attentional allocation^34,35^ derived from difference waves of the electrodes from the hemisphere ipsilateral and contralateral to target presentation, using the PO7 and PO8 occipito-parietal electrodes (Luck, 2012, offers an extensive review in support of this difference wave analysis approach to the N2pc^36^). This way, clear hemispheric differences are lost in the N2pc analyses, and for this reason, N2pc components from both hemispheres are generally combined^36^. Here, this component was assessed according to this approach.

### Statistics

Analyses were performed in the IBM SPSS 20 software. Behavioral (mean reaction times and accuracies) and EEG responses (mean amplitudes and latencies) for the different display types were compared with repeated-measures ANOVAs. These were sphericity checked and Greenhouse-Geisser corrected when appropriate. For the behavioral data, a single ANOVA with the factor Display Type was performed for each outcome measure. For the EEG data, one ANOVA with the two factors (Display Type and Hemisphere) was performed. For the N2pc component, ANOVAs did not include the factor “Hemisphere” as responses at both hemispheres were combined. Tukey HSD post-hoc tests were performed when appropriate.

## Results

### Experiment 1

**Figure 4:**
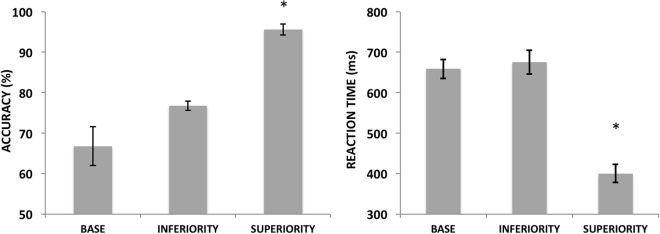
Average accuracies (left) and RTs (right) for the behavioral responses in Experiment 1. Vertical bars represent the standard errors of the mean and conditions for which significant differences were found (i.e. Superiority vs. all the others, p < 0.01) are marked with an asterisk.

For the behavioral data (Fig. 4), repeated measures ANOVAs showed a significant effect of Display Type for both accuracy [F2,30 = 16.68, p < 0.001, ηp^2^ = 0.50] and RT measures [F2,30 = 33.52, p < 0.001, ηp^2^ = 0.69]. Post hoc comparisons showed that in both cases, Superiority was significantly different from Base and Inferiority (p < 0.01). For the P1 ERP, we found a significant effect of Display Type [F2,30 = 6.68, p < 0.01, ηp^2^ = 0.30] and post-hoc tests showed that Superiority was significantly different from Base (p = 0.01) but not from Inferiority (p = 0.7). A borderline significant effect was found for the factor Hemisphere [F1,15 = 3.90, p = 0.06, ηp^2^ = 0.20] and for the interaction between Display Type and Hemisphere [F2,30 = 3.06, p = 0.06, ηp^2^ = 0.16]. An assessment of the pairwise comparisons between all display and hemisphere conditions did not find any relevant and significant comparisons (i.e., interhemispheric difference within the same display type).

**Figure 5:**
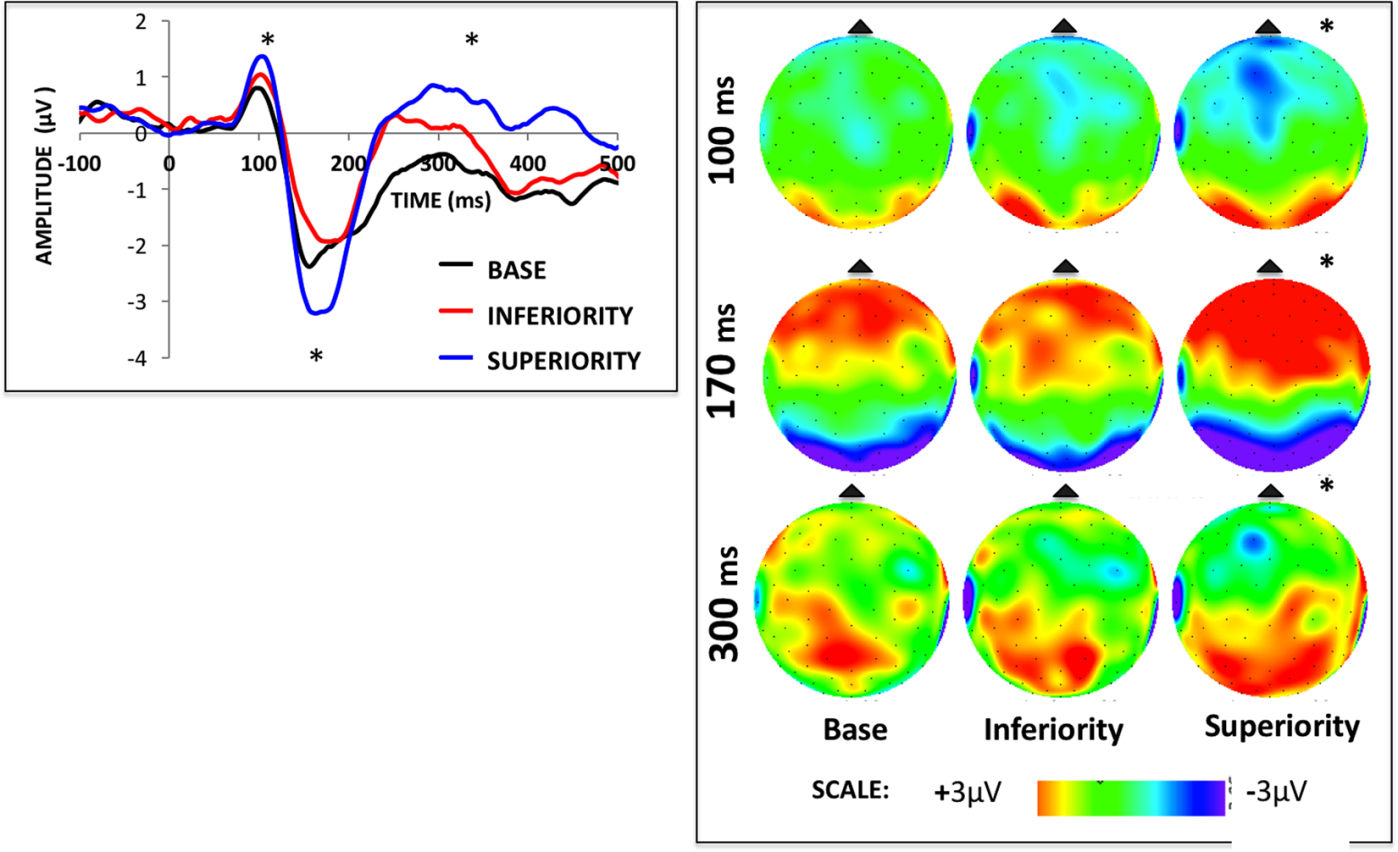
Grand average ERPs (left) and voltage maps for the ERP time windows analyzed (right) in Experiment 1. Voltage maps were created by the linear interpolation of neighboring electrode voltages at each point in time for each stimulus tested, color-coded here from 3µV to −3µV (images show the specific point in time, and not an averaged time window). Conditions that were significantly different from the others are marked with an asterisk (check the text for detailed comparisons).

**Figure 6:**
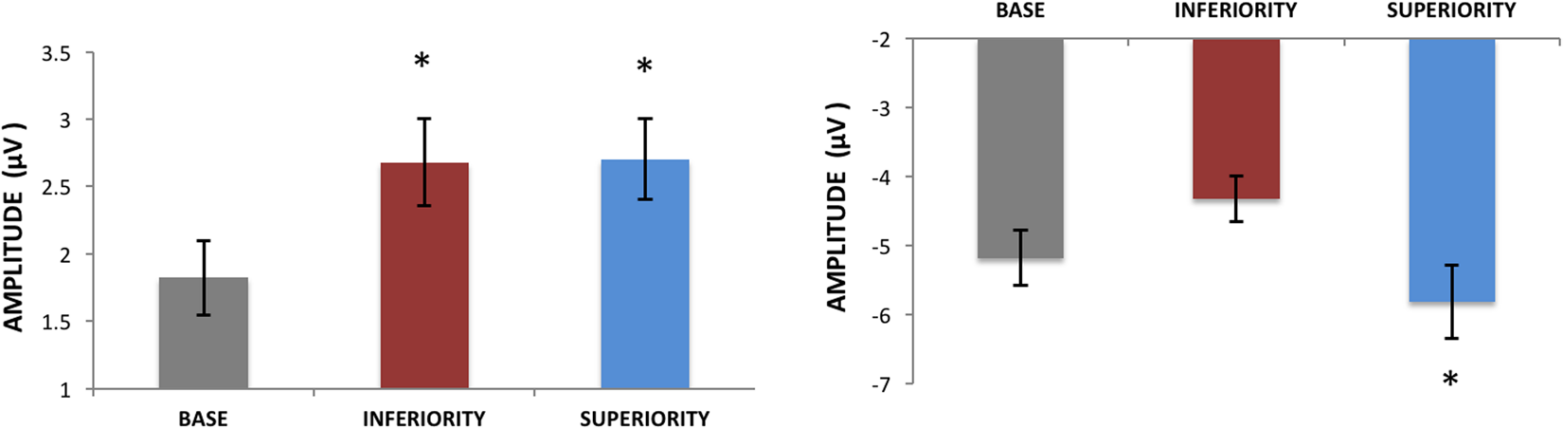
Average amplitudes for the P1 (left) and N1 (right) ERP components in Experiment 1. Vertical bars represent the standard errors of the mean, and conditions for which significant differences were found (p < 0.05) are marked with an asterisk.

For the N1 ERP component (Figs 5 and 6), we found a significant effect of Display Type [F2,30 = 4.65, p < 0.01, ηp^2^ = 0.23], and post-hoc tests showed that Superiority was significantly different from both other display types (p = 0.01). No significant effects were found for the Hemisphere factor [F1,15 = 1.42, p < 0.25, ηp^2^ = 0.08] or the interaction between Display Type and Hemisphere [F2,30 = 0.48, p < 0.92, ηp^2^ < 0.01]. For the late component (peaking between 300 and 400 ms), we found a significant effect of Display Type [F2,30 = 5.56, p < 0.01, ηp^2^ = 0.27], where Superiority was significantly different from both other stimulus conditions (p = 0.01). No significant effects were found for the Hemisphere factor [F1,15 = 1.94, p = 0.18, ηp^2^ = 0.11] or the interaction between Display Type and Hemisphere [F2,30 = 0.68, p < 0.21, ηp^2^ = 0.02].

For the N2pc component amplitude (see figure at the end of the Results section) there was a significant effect of Display Type [F2,30 = 7.3, p < 0.01, ηp^2^ = 0.32] and post hoc analyses showed it was driven by Superiority being different from both other displays (p < 0.01). For the latencies of the N2pc, no such differences were found [F2,30 = 0.69, p < 0.50, ηp^2^ = 0.04].

### Experiment 2

**Figure 7:**
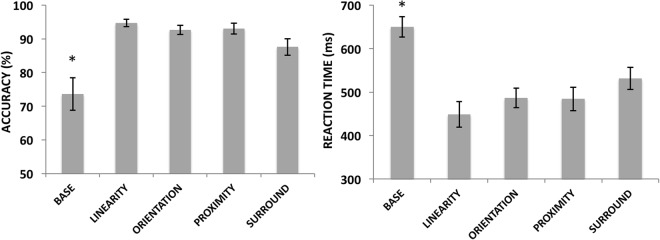
Average accuracies (left) and RTs (right) for the behavioral responses in Experiment 2. Vertical bars represent the standard errors of the mean and conditions that significantly differed from the others (p < 0.01) are marked with an asterisk. In summary, the Base condition differed from all other Display Types for both measures.

Repeated measure ANOVAs for the behavioral data (Fig. 7) showed a significant effect of Display Type [F4,60 = 11.15, p < 0.01, ηp^2^ = 0.42]. Post hoc analyses showed that all Display Types differed from Base (p < 0.01) but not from each other (all p > 0.3). The same trend was seen for the RTs, with a significant effect of Display Type [F4,60 = 15.68, p < 0.01, ηp^2^ = 0.51] and all displays differed from base (p < 0.01) but not from each other (all p > 0.22).

For the P1 component, no significant main effect could be found for Display Type [F4,52 = 0.61, p = 0.65, ηp^2^ = 0.04] or Hemisphere [F1,13 = 0.72, p = 0.41, ηp^2^ = 0.05] alone. However, a significant interaction between Display Type and Hemisphere [F4,52 = 3.07, p = 0.02, ηp^2^ = 0.19] was found. But post hoc analyses did not find any relevant and significant effects (meaning, interhemispheric differences within the same stimulus type or interhemispheric differences between stimulus types).

**Figure 8:**
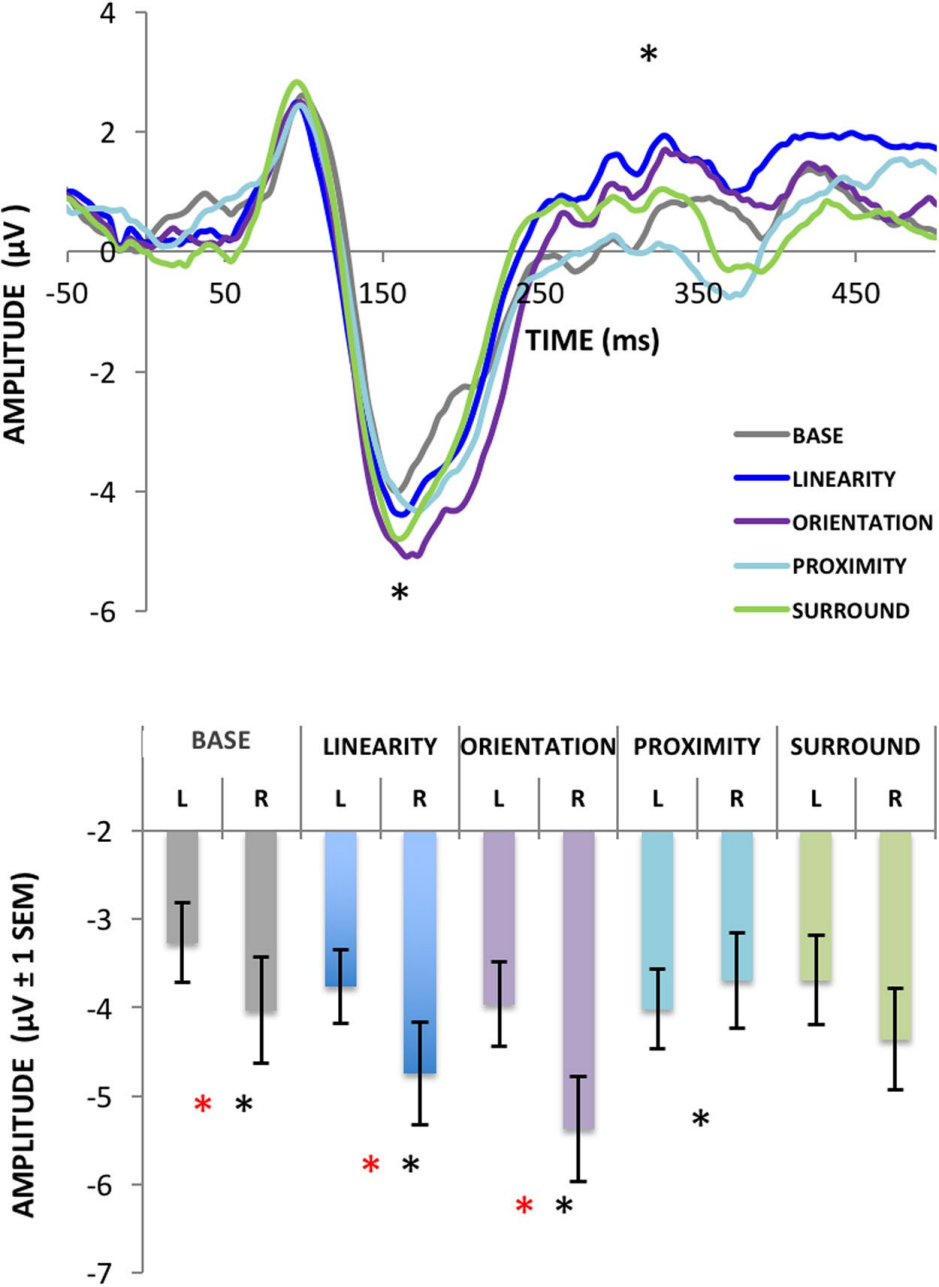
Grand average ERPs (top) and average N1 Peaks (bottom) for Experiment 2. Conditions where a significant hemispheric asymmetry was found are marked with a red asterisk (i.e. Base, Linearity and Orientation with more voltage on the right hemisphere). Conditions that were significantly different from each other within hemisphere are marked with a black asterisk. Linearity and Orientation differed from all other stimuli but not from each other in the right hemisphere. Orientation and Proximity were significantly different from Base in the left hemisphere.

For the N1 component, we found a significant effect of Display Type [F4,60 = 3.45, p < 0.01, ηp^2^ = 0.18], Hemisphere [F1,15 = 5.40, p = 0.03, ηp^2^ = 0.26] and a significant interaction between Display Type and Hemisphere [F4,60 = 9.24, p < 0.01, ηp^2^ = 0.38]. Post hoc analysis found interhemispheric differences for Base (p = 0.01), Orientation (p < 0.01) and Linearity (p < 0.01), shown as increased activity in the right hemisphere in Fig. 8. Proximity and Surroundedness did not differ between hemispheres (p = 0.87 and p = 0.18, respectively). In the within hemisphere post hoc comparisons, Orientation and Linearity on the right hemisphere differed from all other Display Types (p < 0.01), but not from each other (p = 0.10). These EFs did not differ from the other EFs at the left hemisphere. In the left hemisphere, Orientation and Proximity differed from Base (p < 0.05), but not from any of the other Display Types (p < 0.9).

**Figure 9:**
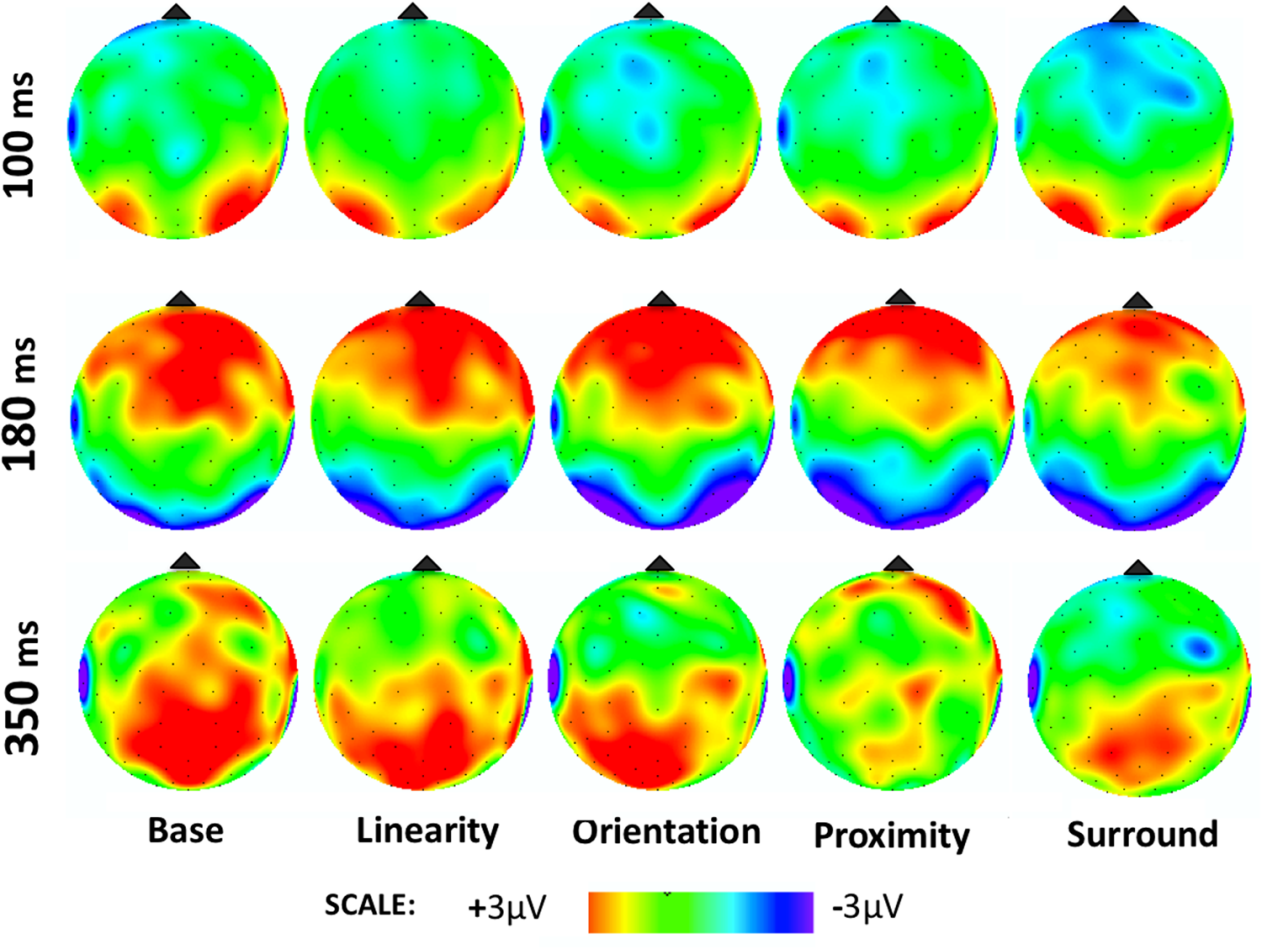
Average voltage maps for the ERP time windows analyzed in Experiment 2. Voltage maps were created by the linear interpolation of neighboring electrode voltages at each point in time for each stimulus tested, color-coded here from 3µV to −3µV (images show the specific point in time, and not averaged time windows).

**Figure 10:**
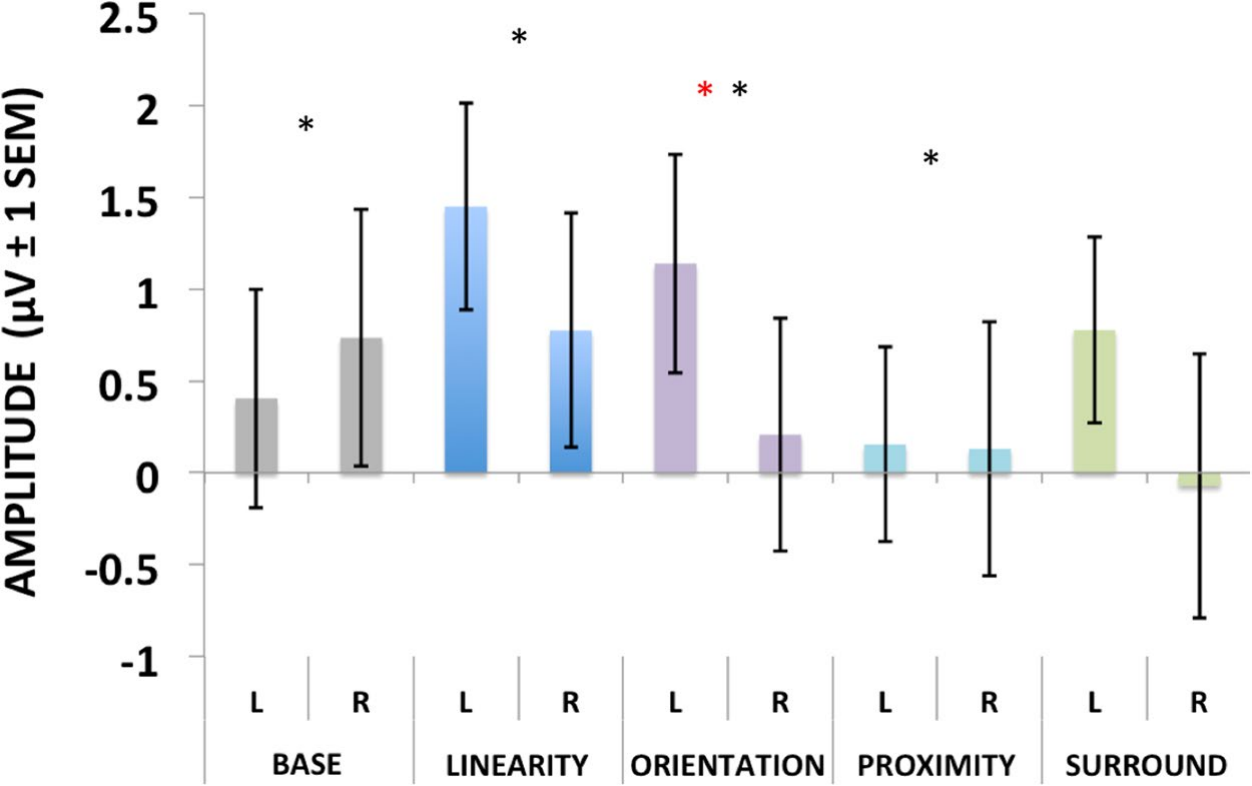
Average ERP voltages for our later component peaking at around 300–400 ms after stimulus onset. Conditions that were significantly different from the others are marked with an asterisk. Conditions where a significant hemispheric asymmetry was found are marked with a red asterisk (check the text for detailed comparisons).

For the late positive component (Fig. 10), there was no effect of Display Type [F4,60 = 1.63, p = 0.17, ηp^2^ = 0.09] or Hemisphere [F1,15 = 1.08, p = 0.31, ηp^2^ = 0.06], but a significant interaction (yet weaker than for the N1) between Display Type and Hemisphere [F4,60 = 4.42, p < 0.01, ηp^2^ = 0.22]. Post hoc analyses showed that only Orientation differed significantly between hemispheres (p < 0.01), and that Linearity and Orientation differed from proximity and Base (p < 0.05), but not from each other (p > 0.5).

**Figure 11:**
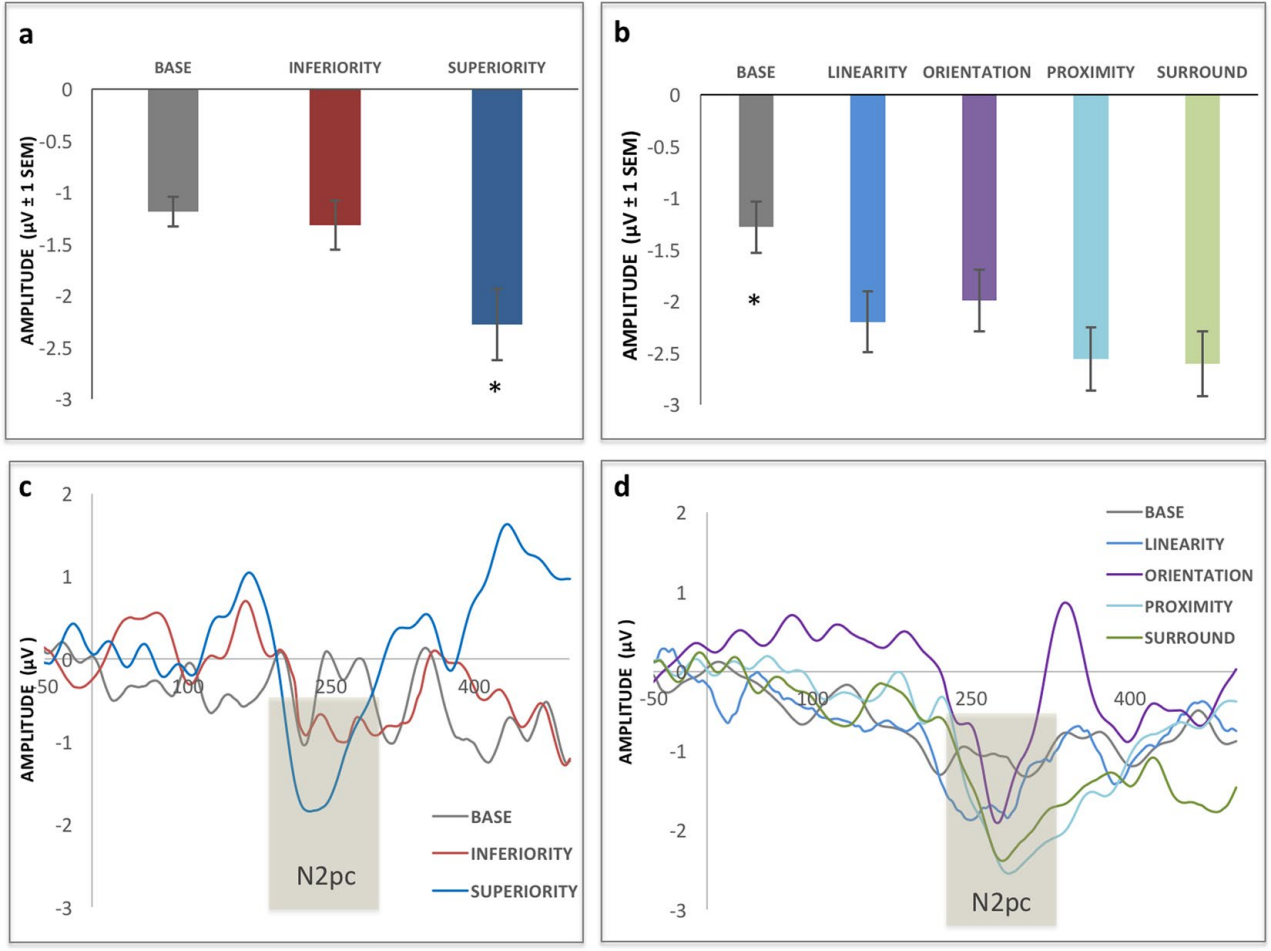
Grand average N2pc for Experiment 1 (left) and Experiment 2 (right). Panels a & b show average peak N2pc amplitudes (vertical bars represent the standard errors of the mean). Conditions that significantly differed from all the others (p < 0.05) are marked with an asterisk (Superiority in Experiment 1 and Base in Experiment 2). Panels c & d show the grand averaged N2pc curves for both hemispheres combined. The time window where the N2pc peaks was scored is marked in gray.

The N2pc component amplitude (Fig. 11) showed a significant effect of Display Type [F4,60 = 4.3, p < 0.01, ηp^2^ = 0.32], and post hoc analyses showed it was driven by Base being different from all other Display Types (p < 0.05). For the latencies of the N2pc, no significant differences were found [F(4,60) = 0.68, p < 0.61, ηp^2^ = 0.04].

## Discussion

At the behavioral level, our results closely replicated the classic findings by Pomerantz *et al*.^3^ and also Pomerantz and Portillo^8^, where adding redundant context elements to a field of targets will aid visual search when EFs are present and impair it (or at least not aid it) when these are missing. In Experiment 2, performance with all stimuli differed significantly from the Base stimulus but not from each other (with either accuracy or RTs). This suggests that the different displays containing EFs that were used here were all equated for saliency, and therefore, the differences found at the level of the ERPs may not be explained in terms of different task difficulty.

In summary, both experiments show significantly different ERPs whenever any of the tested EFs were present. In Experiment 1, displays generating CSEs showed significantly different ERPs from their Base display counterparts as early as the P1 component (although these P1 effects are most likely explained by low-level stimulus features as discussed below). The most remarkable differences emerge at the level of the N1, where CSE-generating stimuli differed from all others. A similar trend was observed in Experiment 2, although now, significant differences started only at the N1 component, and some of these differences were asymmetrically lateralized between brain hemispheres (no hemispheric asymmetries were found for the stimuli containing coexisting multiple EFs in Experiment 1). Most importantly, the results of Experiment 2 suggest that the different EFs assessed here may have different neural correlates. It is not only that the visual selection advantage found in the CSE was associated with larger ERPs across all stimuli tested, but also that these ERPs came from distinct distributions of voltages across the scalp for stimuli composed of different EFs. Each of the three effects mentioned above will be discussed in detail next.

### Increases in ERP amplitudes when EFs are present

One clear trend in our data is that displays that induce CSE were generally associated with increases in ERP amplitude (notably at the N1, N2pc and later). Our results in Experiment 1 suggest that these amplitude increases may not be interpreted as reflecting increased number of stimulus elements in some displays, as Inferiority displays had exactly the same number of lines as Superiority ones and generated clearly different trends (except at the level of the P1, where Superiority and Inferiority were not significantly different from each other). These amplitude increases may have different functional meanings, depending on which ERP component is being measured and peculiarities of the task demands. Here we argue that these might mostly be interpreted either as increased processing demands more generally or as the recruitment of distinct specialized neural populations.

It is not appropriate to simply suggest that these amplitude increases reflect task difficulty for at least two reasons: (i) stimuli in Experiment 2 generated distinct ERPs without significant differences in behavioral responses, and (ii) Vogel & Luck^37^ showed that N1 amplitudes are not affected by task difficulty, arguing against a simple resource-based explanation of N1 and suggesting that it indexes specific discrimination processes. Therefore, we suggest that these amplitude differences reflect neural correlates of emergent configural Gestalts, at least to a great extent.

A whole body of evidence suggests that the N1 is the earliest object selective ERP^38^, with a particular selectivity for faces^39^. Some evidence also suggests that it is differentially sensitive to grouped vs. ungrouped arrays of Gabor stimuli and to the context of these Gabors in a no-report paradigm^29^. But the face selectivity of the N1 is remarkable, leading to a lot of the research on this component to focus on face processing and not so much on perceptual organization. In this context, the results presented here make a strong case for the critical role of this component in the processing of emergent features and configural Gestalts.

In fact, one may also see this face-selectivity of the N1 as reflecting multiple overlapping feature selection processes. This view is highly supported by the lack of consistent differences between different faces (i.e., different identities or emotional expressions) at the level of the N1^40^. Consistent face-related changes in the N1 are generally found only when a face is devoid of some of its features or inverted^41^. A more recent review by Rossion^42^ may support (at least in part) this account of the N1. It highlights evidence of the existence of multiple concurrent processing steps in the N1 time window that would gradually allow for a coarse to fine-grained representation of face stimuli. We argue here that the differential N1 modulation by different EFs is further evidence of these overlapping processes. But as we will discuss below, it is also remarkable that our N1 effects were somewhat lateralized.

### Hemispheric asymmetries in the processing of EFs

As mentioned above, it is clear from previous studies that the N1 component is sensitive to grouped vs. ungrouped contours and is particularly object-selective. However, to our knowledge there is no other report on the selectivity of N1 to differential EFs in terms of hemispheric asymmetries. In Experiment 2, when isolating different EFs, we have found that different patterns of voltage distributions were found across the scalp, asymmetrically for the two hemispheres (Figs 8 and 9). The literature lacks assessments of potential functional asymmetries of different brain hemispheres in the processing of different EFs, but has investigated functional asymmetries in the processing of global vs. local stimulus features with somewhat controversial findings so far. This literature is also critical here as it could be argued that there are substantially more demands for local visual processing in the Base displays used in both of our experiments (single dots of lines) and more global demands for the displays possessing clear EFs.

It is well established that hemispheric asymmetries are a critical trait of brain organization across many species^43^. When it comes to functional asymmetries in the human visual brain, a few neuropsychological findings with brain-lesioned patients have suggested that right hemisphere is specialized for the processing of global image features, while the left hemisphere is specialized for the processing of local elements^44^. However, studies with healthy participants and multiple techniques (ranging from psychophysics to neuroimaging) have found a less clear-cut separation of these functions across hemispheres, if any at all^45^.

Some of the contradictory findings in literature are hard to reconcile, but many authors have proposed that other hemispheric specializations may be acting as confounding factors here, driving some of the contradictory effects in literature. As reviewed by Kimchi^46,47^, there are two remarkable arguments in this direction: the potential hemispheric specialization for different spatial frequency content^48^ or for relative saliency^49,50^.

There is indeed evidence for hemispheric specialization for different spatial frequency content (with the left hemisphere dealing with high and the right with low spatial frequencies), and these may be critical to explain part of the global-local hemispheric asymmetries in experiments using compound letter or number stimuli, as relative feature sizes are different in these stimuli (with the local features possessing more high spatial frequency content than the global ones). Some studies have equated local and global features in terms of their spatial frequency content and found that the hemispheric asymmetries have indeed decreased^51^, but spatial frequency content seems to not be able to explain all the results in this field^45,52^.

There is also the debate on whether relative saliency of features could account for some of the hemispheric asymmetries in the processing of global vs. local features^49,50,53^. Hübner and Volberg^54^ have assessed this issue and proposed that both hemispheres process local level features similarly but differ in the specialization to bind these features hierarchically. This idea also finds some support in other works^55^, but there is no consensus on this interpretation, and the issue is far from being resolved.

In the case of the results found in Experiment 2, neither relative saliency nor spatial frequency seem to be potential explanations behind the hemispheric asymmetries found (as different EFs were equated for saliency and had equivalent spatial frequency content too). It is also hard to argue that different EF displays used (e.g., orientation vs. proximity) differ in their demands for global or local visual processing. In fact, the only systematic differences between the displays were the EFs (the number of elements in different displays is different, but increases in ERP amplitudes did not mirror increases in number of elements in either Experiment 1 or 2). When multiple EFs were presented together in Experiment 1 (for the displays inducing a CSE), no hemispheric asymmetries were found (as seemed to be the case in other studies using similar stimuli in an fMRI setup^23,25^). However, when we isolated different EFs in Experiment 2, these asymmetries were clear, potentially making a case for how different EFs might be processed with different weights in different hemispheres. In support of this hypothesis, Orientation and Linearity did not differ significantly from each other. This may suggest that both rely on similar mechanisms of global orientation processing (not to be confused with the specific orientation selectivity of V1 cells, as the processes mentioned here would involve multiple levels of processing), in accordance with the hypothesis that these EFs may be related to each other in a somewhat hierarchical way^8^.

In sum, these results challenge a more simplistic view of hemispheric asymmetries in the processing of hierarchical image structure or global vs. local feature selection in the visual system. It suggests that the processing of different EFs may be significantly lateralized at specific points in time and that these differences may be captured at the level of ERPs. Not many studies have employed an approach similar to the one used here (i.e., allowing for comparing different EFs in the same common scale with minimal confounders) in a neuroimaging setup or with neuropsychological patients. Consequently, our understanding of potential hemispheric asymmetries in the processing of different EFs is still limited, and we argue that our results provide a critical step forward.

### Feature-based attentional selection and N2Pc

Since we employed a task where known features are expected at unknown locations (as different display types were blocked together, but the location of the target in the odd-quadrant display was random), feature-based attention and selection play a major role in performance. The N2pc component is a well-established marker of such processes^34^. One might expect EFs and the CSE to be associated with differential engagement of the feature-based attentional mechanisms signaled by the N2pc. This is exactly what was found. However, given the strong RT differences between conditions (up to 250 ms on average), it is surprising that these differences were only visible in terms of amplitude, not latency of the ERPs.

We argue that the different number of local elements in each display is not a significant confounder here. Mazza *et al*.^56^ assessed the functional significance of the N2pc, finding that a number of manipulations (such as the number, heterogeneity, or spatial proximity of distractors) affected RTs but did not affect N2pc amplitude. According to this, different RTs and different demands for the different display types tested here are unable to explain the enhanced N2pc amplitude found when EFs are present. But the enhancement of N2pc found here is in accordance with the hypothesis of Mazza *et al*.^56^ (and others^57,58^) that the N2pc is a neural signature of feature-based selection and not of attentional filtering and distractor suppression.

For all stimuli tested in Experiment 1 and 2, task demands were always the same (look for an odd element in an array of four items), and the only difference between conditions was the presence or absence of different EFs in some of the targets. These results then support a particular feature-based attentional allocation mechanism for when EFs are present. The few studies that assessed the sources of the N2pc component have yielded compelling evidence for how, in the ventral visual pathway, attention to features may precede attention to specific locations^59^, spreading from the lateral occipital complex and including areas like V4^60^. Recalling the results of the two fMRI studies that have assessed the neural correlates of the CSE^23,25^, both suggest stronger responses on the shape-selective lateral occipital cortex when stimuli lead to a CSE. Our N2pc and N1 results support the idea that these effects are present in the 100–200 ms time window after stimulus onset.

## Conclusions

The results presented here suggest that different EFs are related to different EEG signatures. These also suggest that the Configural Superiority Effect is driven by processes present in the time window between 100 ms and 200 ms after stimulus onset, and that this effect may be different depending on which EFs are present in the stimulus set tested. It makes a case for the specificity (and not the generality) of the neural correlates of different EFs (and therefore, different grouping principles). Experiments 1 and 2 challenge a more simplistic view of N1 as broad feature selective mechanism and adds to the body of knowledge on hemispheric asymmetries on the processing of hierarchical image structure. It also suggests that particular attention allocation mechanisms are engaged when EFs are present, adding to the body of literature on feature-based attentional selection of good Gestalt features. All these effects were strong enough to be assessed at the level of ERPs. This supports that the approach used here is robust in terms of isolating neural correlates of different EFs and can be adapted for future investigations of these issues either in healthy or in clinical populations.

## Data Availability

The datasets generated during and/or analyzed during the current study are available from the corresponding author on reasonable request.
